# {*N*′-[(*E*)-1-(5-Chloro-2-oxidophen­yl)ethyl­idene]-4-meth­oxy­benzohydrazidato-κ^3^ *O*,*N*′,*O*′}(1*H*-imidazole-κ*N* ^3^)nickel(II)

**DOI:** 10.1107/S2414314622002954

**Published:** 2022-03-22

**Authors:** Jian-Guo Chang

**Affiliations:** a Department of Chemistry and Chemical Engineering, Taishan University, 271021 Taian, Shandong, People’s Republic of China; Sunway University, Malaysia

**Keywords:** crystal structure, acyl­hydrazone, hydrogen bonding, imidazole, nickel complex

## Abstract

In the title complex, [Ni(C_16_H_13_ClN_2_O_3_)(C_3_H_4_N_2_)], the Ni^II^ ion is coordinated by two O atoms and one N atom derived from the dianionic *N*′-[(1*E*)-1-(5-chloro-2-hy­droxy­phen­yl)ethyl­idene]-4-meth­oxy­benzohydrazide ligand and one N atom from the imidazole mol­ecule. The N_2_O_2_ donor set defines an approximate square-planar geometry. The dihedral angles between the imidazole ring and the fused six-membered and meth­oxy­benzene rings are 17.78 (14) and 13.23 (16)°, respectively; the dihedral angle between the C_6_ rings is 6.63 (12)°. The most prominent feature of the mol­ecular packing is the formation of 4_1_-helical chains (along the *c* axis) mediated by imidazole-N—H⋯O(phenoxide) hydrogen bonding; these are linked by methyl-C—H⋯Cl inter­actions.

## Abstract 2

The title complex displays a slightly distorted square-planar coordination geometry. The crystal features 4_1_-helical chains stabilized by N—H⋯O hydrogen bonding.

## Graphical abstract

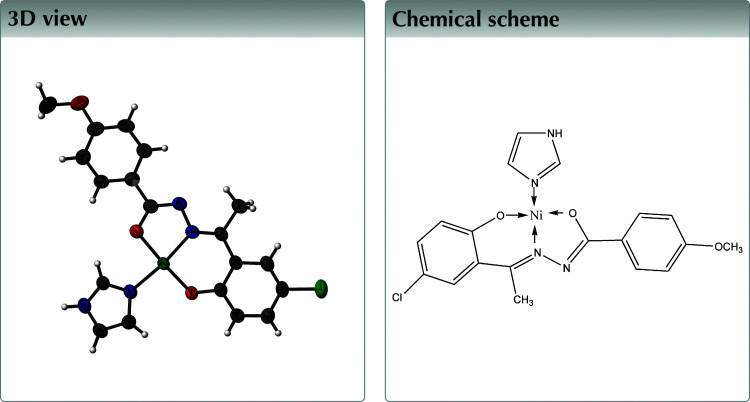

## Structure description

Acyl­hydrazones, as a special kind of Schiff base, have been widely investigated because of their strong coordination ability (Singh *et al.*, 1982 ▸; Salem, 1998 ▸; Yu *et al.*, 2010 ▸) and flexible coordination modes involving the N and O donor atoms (Liu *et al.*, 2005 ▸; Chang, 2011 ▸; Zheng *et al.*, 2011 ▸). As has been widely reported in the literature, acyl­hydrazone complexes display various biological activities such as anti-microbial (Yang *et al.*, 2020 ▸), anti-tubercular (Peng, 2011 ▸), anti-cancer (Morgan *et al.*, 2003 ▸) and anti-oxidant (Chang *et al.*, 2015 ▸). As an extension of work into the structural characterization of aroylhydrazone complexes, the title complex, [Ni(C_16_H_13_ClN_2_O_3_)(C_3_H_4_N_2_)], has been synthesized and its crystal structure determined.

**Figure 1:**
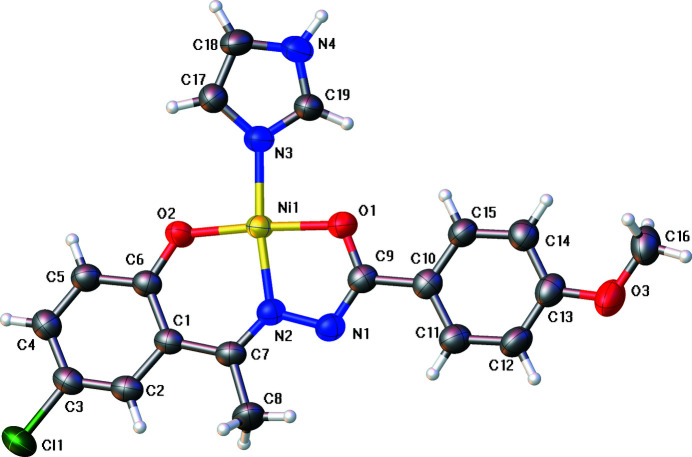
The mol­ecular structure, showing the atom-numbering scheme. Displacement ellipsoids are drawn at the 50% probability level.

The Ni^II^ ion in the title compound is coordinated by two O atoms and one N atom from the dianionic *N*′-[(1*E*)-1-(5-chloro-2-hy­droxy­phen­yl)ethyl­idene]-4-meth­oxy­benzo­hy­dra­zide ligand and one N atom from the imidazole mol­ecule. In this complex, the Ni atom is located in a slightly distorted square-planar environment (Fig. 1 ▸ and Table 1 ▸). The Ni—O bond lengths are systematically shorter than the Ni—N bonds, and the maximum deviation from the ideal square-planar geometry in terms of angles is found for O1—Ni1—N2 = 82.18 (7)°. The two benzene rings, C1–C6 (*A*) and C10–C15 (*B*), and the imidazole ring (*C*) make dihedral angles of 6.63 (12)° (*A*/*B*), 17.78 (14)° (*A*/*C*) and 13.23 (16)° (*B*/*C*).

**Figure 2:**
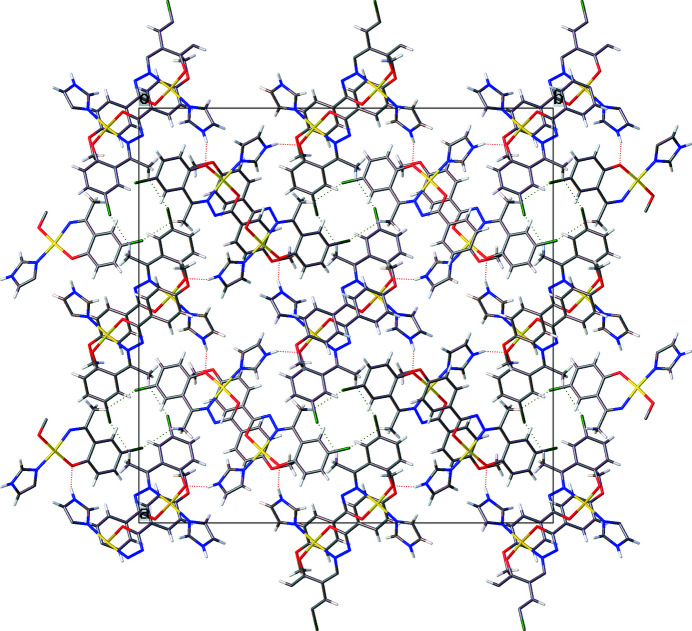
A view in projection down the *c* axis of the unit-cell contents.

The mol­ecular packing is consolidated by imidazole-N—H⋯O(phenoxide) hydrogen bonding (Table 2 ▸) along the *c* axis, which leads to a 4_1_ helical chain. The chains are connected by C—H⋯Cl inter­actions (Table 2 ▸) into a three-dimensional architecture; a view of the unit-cell contents is given in Fig. 2 ▸.

## Synthesis and crystallization

The Schiff base ligand, *N*′-[(1*E*)-1-(5-chloro-2-hy­droxyphen­yl)ethyl­idene]-4-meth­oxy­benzohydrazide (0.100 mmol, 0.0319 g), 1*H*-imidazole (0.100 mmol, 0.0068 g), Ni(NO_3_)_2_·6H_2_O (0.100 mmol, 0.0292 g), methanol (10 ml) and distilled water (5 ml) were mixed in a 50 ml flask. The mixture was stirred at room temperature for 1 h, the pH was adjusted with saturated sodium carbonate solution to about 8 followed by filtration. Red rectangular block-shaped crystals were obtained after about one month by evaporating the filtrate in air (yield 31%).

## Refinement

Crystal data, data collection and structure refinement details are summarized in Table 3 ▸.

## Supplementary Material

Crystal structure: contains datablock(s) I. DOI: 10.1107/S2414314622002954/tk4076sup1.cif

Structure factors: contains datablock(s) I. DOI: 10.1107/S2414314622002954/tk4076Isup2.hkl

CCDC reference: 2159183

Additional supporting information:  crystallographic information; 3D view; checkCIF report

## Figures and Tables

**Table 1:** Selected geometric parameters (Å, °)

Ni1—O1	1.8995 (15)	Ni1—N2	1.9344 (17)
Ni1—O2	1.8820 (15)	Ni1—N3	1.9664 (18)
			
O1—Ni1—N2	82.18 (7)	O2—Ni1—N2	94.33 (7)
O1—Ni1—N3	90.33 (7)	O2—Ni1—N3	93.16 (7)
O2—Ni1—O1	173.63 (7)	N2—Ni1—N3	172.50 (8)

**Table 2:** Hydrogen-bond geometry (Å, °)

*D*—H⋯*A*	*D*—H	H⋯*A*	*D*⋯*A*	*D*—H⋯*A*
N4—H4⋯O2^i^	0.92 (4)	1.93 (4)	2.818 (3)	161 (3)
C8—H8*A*⋯Cl1^ii^	0.96	2.86	3.700 (3)	147

Symmetry codes: (i) 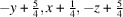 ; (ii) 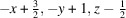 .

**Table 3:** Experimental details

Crystal data	
Chemical formula	[Ni(C_16_H_13_ClN_2_O_3_)(C_3_H_4_N_2_)]
*M*_r_	443.52
Crystal system, space group	Tetragonal, *I*4_1_/*a*
Temperature (K)	295
*a*, *c* (Å)	30.879 (4), 8.0750 (16)
*V* (Å^3^)	7700 (3)
*Z*	16
Radiation type	Mo *K*α
μ (mm^−1^)	1.18
Crystal size (mm)	0.35 × 0.25 × 0.16

Data collection	
Diffractometer	Bruker APEXII CCD area detector
Absorption correction	Multi-scan (*SADABS*; Bruker, 2003 ▸)
*T*_min_, *T*_max_	0.735, 0.860
No. of measured, independent and observed [*I* > 2σ(*I*)] reflections	23938, 4924, 3431
*R*_int_	0.038
(sin θ/λ)_max_ (Å^−1^)	0.678

Refinement	
*R*[*F* ^2^ > 2σ(*F* ^2^)], *wR*(*F* ^2^), *S*	0.038, 0.106, 1.00
No. of reflections	4924
No. of parameters	259
H-atom treatment	H atoms treated by a mixture of independent and constrained refinement
Δρ_max_, Δρ_min_ (e Å^−3^)	0.79, −0.21

Computer programs: *APEX2* and *SAINT* (Bruker, 2003 ▸), *SHELXT* (Sheldrick, 2015*a* ▸), *SHELXL* (Sheldrick, 2015*b* ▸) and *OLEX2* (Dolomanov *et al.*, 2009 ▸).

## full crystallographic data

### Crystal data

**Table:**

[Ni(C_16_H_13_ClN_2_O_3_)(C_3_H_4_N_2_)]	*D*_x_ = 1.530 Mg m^−^^3^
*M**_r_* = 443.52	Mo *K*α radiation, λ = 0.71073 Å
Tetragonal, *I*4_1_/*a*	Cell parameters from 5154 reflections
*a* = 30.879 (4) Å	θ = 2.6–25.1°
*c* = 8.0750 (16) Å	µ = 1.18 mm^−^^1^
*V* = 7700 (3) Å^3^	*T* = 295 K
*Z* = 16	Block, brown
*F*(000) = 3648	0.35 × 0.25 × 0.16 mm

### Data collection

**Table:**

Bruker APEXII CCD area detector diffractometer	3431 reflections with *I* > 2σ(*I*)
Graphite monochromator	*R*_int_ = 0.038
phi and ω scans	θ_max_ = 28.8°, θ_min_ = 1.9°
Absorption correction: multi-scan (SADABS; Bruker, 2003)	*h* = −28→40
*T*_min_ = 0.735, *T*_max_ = 0.860	*k* = −41→39
23938 measured reflections	*l* = −10→10
4924 independent reflections	

### Refinement

**Table:**

Refinement on *F*^2^	Primary atom site location: dual
Least-squares matrix: full	Hydrogen site location: mixed
*R*[*F*^2^ > 2σ(*F*^2^)] = 0.038	H atoms treated by a mixture of independent and constrained refinement
*wR*(*F*^2^) = 0.106	*w* = 1/[σ^2^(*F*_o_^2^) + (0.0616*P*)^2^] where *P* = (*F*_o_^2^ + 2*F*_c_^2^)/3
*S* = 1.00	(Δ/σ)_max_ = 0.001
4924 reflections	Δρ_max_ = 0.79 e Å^−^^3^
259 parameters	Δρ_min_ = −0.20 e Å^−^^3^
0 restraints	

### Special details

**Table:**

Geometry. All esds (except the esd in the dihedral angle between two l.s. planes) are estimated using the full covariance matrix. The cell esds are taken into account individually in the estimation of esds in distances, angles and torsion angles; correlations between esds in cell parameters are only used when they are defined by crystal symmetry. An approximate (isotropic) treatment of cell esds is used for estimating esds involving l.s. planes.

### Fractional atomic coordinates and isotropic or equivalent isotropic displacement parameters (Å^2^)

**Table:**

	*x*	*y*	*z*	*U*_iso_*/*U*_eq_	
Ni1	0.67356 (2)	0.70309 (2)	0.69948 (3)	0.03734 (10)	
Cl1	0.68036 (2)	0.49257 (2)	1.08616 (9)	0.06233 (19)	
O1	0.71130 (5)	0.74129 (5)	0.5847 (2)	0.0506 (4)	
O2	0.63727 (4)	0.66083 (5)	0.7940 (2)	0.0468 (4)	
O3	0.86943 (7)	0.82096 (6)	0.1939 (3)	0.0818 (6)	
N1	0.75900 (5)	0.68423 (6)	0.6093 (2)	0.0444 (4)	
N2	0.72374 (5)	0.66565 (5)	0.6917 (2)	0.0407 (4)	
N3	0.62734 (6)	0.74687 (6)	0.6931 (2)	0.0469 (4)	
N4	0.59505 (7)	0.80650 (7)	0.6168 (3)	0.0573 (5)	
H4	0.5865 (10)	0.8318 (11)	0.566 (4)	0.100 (11)*	
C1	0.69302 (6)	0.60618 (6)	0.8443 (3)	0.0395 (5)	
C2	0.70039 (8)	0.56518 (7)	0.9178 (3)	0.0468 (5)	
H2	0.728018	0.553255	0.912614	0.056*	
C3	0.66852 (8)	0.54265 (7)	0.9955 (3)	0.0471 (5)	
C4	0.62689 (8)	0.55887 (7)	1.0092 (3)	0.0494 (5)	
H4A	0.605335	0.543496	1.064097	0.059*	
C5	0.61852 (7)	0.59863 (7)	0.9385 (3)	0.0478 (5)	
H5	0.590670	0.609875	0.946780	0.057*	
C6	0.64995 (7)	0.62290 (7)	0.8548 (3)	0.0406 (5)	
C7	0.72926 (7)	0.62824 (7)	0.7636 (3)	0.0418 (5)	
C8	0.77365 (8)	0.60794 (9)	0.7632 (4)	0.0637 (7)	
H8A	0.772858	0.581362	0.701572	0.095*	
H8B	0.782430	0.602062	0.875021	0.095*	
H8C	0.793945	0.627451	0.712772	0.095*	
C9	0.74864 (7)	0.72329 (7)	0.5599 (3)	0.0430 (5)	
C10	0.78074 (7)	0.74904 (7)	0.4658 (3)	0.0436 (5)	
C11	0.82214 (7)	0.73375 (8)	0.4304 (3)	0.0512 (6)	
H11	0.830661	0.706668	0.468830	0.061*	
C12	0.85065 (8)	0.75850 (8)	0.3386 (3)	0.0585 (6)	
H12	0.878025	0.747719	0.313970	0.070*	
C13	0.83883 (8)	0.79913 (8)	0.2832 (3)	0.0544 (6)	
C14	0.79796 (8)	0.81495 (8)	0.3173 (3)	0.0531 (6)	
H14	0.789633	0.842107	0.279077	0.064*	
C15	0.76968 (7)	0.79008 (7)	0.4085 (3)	0.0487 (5)	
H15	0.742350	0.801047	0.432655	0.058*	
C16	0.85944 (10)	0.86421 (8)	0.1450 (4)	0.0709 (8)	
H16A	0.836204	0.863811	0.066026	0.106*	
H16B	0.884538	0.877267	0.095671	0.106*	
H16C	0.850860	0.880669	0.240361	0.106*	
C17	0.58978 (7)	0.75137 (7)	0.7827 (3)	0.0517 (6)	
H17	0.579704	0.732134	0.862486	0.062*	
C18	0.56989 (8)	0.78836 (8)	0.7357 (4)	0.0587 (6)	
H18	0.543965	0.799272	0.776902	0.070*	
C19	0.62897 (8)	0.78101 (7)	0.5950 (3)	0.0527 (6)	
H19	0.651171	0.786450	0.519968	0.063*	

### Atomic displacement parameters (Å^2^)

**Table:**

	*U*^11^	*U*^22^	*U*^33^	*U*^12^	*U*^13^	*U*^23^
Ni1	0.03354 (15)	0.03200 (15)	0.04649 (18)	0.00236 (10)	−0.00014 (11)	−0.00031 (11)
Cl1	0.0762 (4)	0.0409 (3)	0.0699 (4)	0.0076 (3)	−0.0036 (3)	0.0093 (3)
O1	0.0434 (8)	0.0419 (8)	0.0666 (11)	0.0041 (7)	0.0079 (7)	0.0037 (7)
O2	0.0370 (8)	0.0403 (8)	0.0633 (10)	0.0022 (6)	−0.0021 (7)	0.0089 (7)
O3	0.0736 (13)	0.0594 (11)	0.1126 (18)	−0.0087 (10)	0.0426 (12)	0.0024 (11)
N1	0.0367 (9)	0.0453 (10)	0.0512 (11)	−0.0002 (8)	−0.0009 (8)	−0.0029 (8)
N2	0.0367 (9)	0.0404 (9)	0.0451 (10)	−0.0002 (7)	−0.0013 (7)	−0.0040 (8)
N3	0.0440 (10)	0.0410 (10)	0.0557 (12)	0.0050 (8)	0.0017 (8)	0.0010 (8)
N4	0.0515 (12)	0.0400 (11)	0.0804 (16)	0.0071 (9)	0.0033 (11)	0.0071 (10)
C1	0.0424 (11)	0.0376 (11)	0.0387 (11)	0.0045 (9)	−0.0046 (9)	−0.0029 (9)
C2	0.0489 (12)	0.0433 (12)	0.0484 (13)	0.0082 (10)	−0.0056 (10)	−0.0024 (10)
C3	0.0566 (13)	0.0373 (11)	0.0473 (12)	0.0045 (10)	−0.0057 (10)	−0.0007 (9)
C4	0.0540 (13)	0.0441 (12)	0.0502 (13)	−0.0020 (10)	−0.0010 (11)	0.0026 (10)
C5	0.0412 (12)	0.0456 (12)	0.0565 (15)	0.0023 (9)	−0.0021 (10)	0.0009 (10)
C6	0.0405 (11)	0.0393 (11)	0.0420 (12)	0.0018 (9)	−0.0058 (9)	−0.0013 (9)
C7	0.0400 (11)	0.0418 (11)	0.0435 (12)	0.0069 (9)	−0.0056 (9)	−0.0027 (9)
C8	0.0442 (13)	0.0688 (16)	0.0781 (19)	0.0146 (12)	0.0061 (12)	0.0174 (14)
C9	0.0408 (11)	0.0439 (12)	0.0445 (13)	−0.0017 (9)	−0.0017 (9)	−0.0078 (9)
C10	0.0429 (12)	0.0463 (12)	0.0417 (12)	−0.0041 (9)	−0.0024 (9)	−0.0076 (9)
C11	0.0454 (12)	0.0461 (13)	0.0620 (15)	−0.0011 (10)	0.0004 (11)	−0.0066 (11)
C12	0.0408 (12)	0.0607 (15)	0.0741 (18)	0.0006 (11)	0.0106 (12)	−0.0041 (13)
C13	0.0504 (13)	0.0525 (14)	0.0602 (16)	−0.0101 (10)	0.0098 (11)	−0.0101 (11)
C14	0.0566 (14)	0.0463 (13)	0.0566 (15)	−0.0028 (11)	0.0043 (11)	−0.0021 (11)
C15	0.0422 (12)	0.0495 (13)	0.0545 (14)	0.0021 (10)	0.0060 (10)	−0.0012 (10)
C16	0.0801 (19)	0.0578 (16)	0.0749 (19)	−0.0166 (14)	0.0204 (15)	−0.0005 (14)
C17	0.0507 (13)	0.0438 (12)	0.0604 (15)	0.0005 (10)	0.0077 (11)	0.0029 (11)
C18	0.0496 (14)	0.0459 (13)	0.0805 (18)	0.0100 (11)	0.0115 (13)	−0.0003 (12)
C19	0.0485 (13)	0.0432 (12)	0.0665 (16)	0.0074 (10)	0.0089 (11)	0.0090 (11)

### Geometric parameters (Å, º)

**Table:**

Ni1—O1	1.8995 (15)	C5—H5	0.9300
Ni1—O2	1.8820 (15)	C5—C6	1.400 (3)
Ni1—N2	1.9344 (17)	C7—C8	1.507 (3)
Ni1—N3	1.9664 (18)	C8—H8A	0.9600
Cl1—C3	1.750 (2)	C8—H8B	0.9600
O1—C9	1.296 (2)	C8—H8C	0.9600
O2—C6	1.329 (2)	C9—C10	1.481 (3)
O3—C13	1.367 (3)	C10—C11	1.392 (3)
O3—C16	1.426 (3)	C10—C15	1.392 (3)
N1—N2	1.399 (2)	C11—H11	0.9300
N1—C9	1.310 (3)	C11—C12	1.381 (3)
N2—C7	1.304 (3)	C12—H12	0.9300
N3—C17	1.374 (3)	C12—C13	1.381 (3)
N3—C19	1.320 (3)	C13—C14	1.381 (3)
N4—H4	0.92 (4)	C14—H14	0.9300
N4—C18	1.356 (3)	C14—C15	1.376 (3)
N4—C19	1.322 (3)	C15—H15	0.9300
C1—C2	1.417 (3)	C16—H16A	0.9600
C1—C6	1.429 (3)	C16—H16B	0.9600
C1—C7	1.463 (3)	C16—H16C	0.9600
C2—H2	0.9300	C17—H17	0.9300
C2—C3	1.359 (3)	C17—C18	1.351 (3)
C3—C4	1.384 (3)	C18—H18	0.9300
C4—H4A	0.9300	C19—H19	0.9300
C4—C5	1.378 (3)		
			
O1—Ni1—N2	82.18 (7)	C7—C8—H8B	109.5
O1—Ni1—N3	90.33 (7)	C7—C8—H8C	109.5
O2—Ni1—O1	173.63 (7)	H8A—C8—H8B	109.5
O2—Ni1—N2	94.33 (7)	H8A—C8—H8C	109.5
O2—Ni1—N3	93.16 (7)	H8B—C8—H8C	109.5
N2—Ni1—N3	172.50 (8)	O1—C9—N1	124.4 (2)
C9—O1—Ni1	110.80 (13)	O1—C9—C10	116.41 (19)
C6—O2—Ni1	125.85 (13)	N1—C9—C10	119.18 (19)
C13—O3—C16	117.3 (2)	C11—C10—C9	122.5 (2)
C9—N1—N2	109.43 (17)	C15—C10—C9	119.69 (19)
N1—N2—Ni1	113.18 (12)	C15—C10—C11	117.8 (2)
C7—N2—Ni1	128.30 (15)	C10—C11—H11	119.7
C7—N2—N1	118.25 (17)	C12—C11—C10	120.5 (2)
C17—N3—Ni1	131.96 (16)	C12—C11—H11	119.7
C19—N3—Ni1	122.48 (16)	C11—C12—H12	119.7
C19—N3—C17	105.51 (18)	C13—C12—C11	120.5 (2)
C18—N4—H4	120 (2)	C13—C12—H12	119.7
C19—N4—H4	132 (2)	O3—C13—C12	115.9 (2)
C19—N4—C18	107.6 (2)	O3—C13—C14	124.3 (2)
C2—C1—C6	116.57 (19)	C14—C13—C12	119.9 (2)
C2—C1—C7	118.67 (18)	C13—C14—H14	120.4
C6—C1—C7	124.76 (18)	C15—C14—C13	119.3 (2)
C1—C2—H2	118.8	C15—C14—H14	120.4
C3—C2—C1	122.3 (2)	C10—C15—H15	119.0
C3—C2—H2	118.8	C14—C15—C10	122.0 (2)
C2—C3—Cl1	119.64 (17)	C14—C15—H15	119.0
C2—C3—C4	121.6 (2)	O3—C16—H16A	109.5
C4—C3—Cl1	118.72 (18)	O3—C16—H16B	109.5
C3—C4—H4A	121.2	O3—C16—H16C	109.5
C5—C4—C3	117.6 (2)	H16A—C16—H16B	109.5
C5—C4—H4A	121.2	H16A—C16—H16C	109.5
C4—C5—H5	118.4	H16B—C16—H16C	109.5
C4—C5—C6	123.1 (2)	N3—C17—H17	125.6
C6—C5—H5	118.4	C18—C17—N3	108.8 (2)
O2—C6—C1	124.79 (19)	C18—C17—H17	125.6
O2—C6—C5	116.47 (18)	N4—C18—H18	126.6
C5—C6—C1	118.72 (19)	C17—C18—N4	106.7 (2)
N2—C7—C1	120.71 (18)	C17—C18—H18	126.6
N2—C7—C8	119.1 (2)	N3—C19—N4	111.4 (2)
C1—C7—C8	120.19 (19)	N3—C19—H19	124.3
C7—C8—H8A	109.5	N4—C19—H19	124.3
			
Ni1—O1—C9—N1	−0.8 (3)	C2—C1—C7—C8	−1.5 (3)
Ni1—O1—C9—C10	177.82 (14)	C2—C3—C4—C5	1.0 (3)
Ni1—O2—C6—C1	−6.1 (3)	C3—C4—C5—C6	0.0 (3)
Ni1—O2—C6—C5	172.09 (15)	C4—C5—C6—O2	−179.5 (2)
Ni1—N2—C7—C1	9.6 (3)	C4—C5—C6—C1	−1.2 (3)
Ni1—N2—C7—C8	−170.21 (18)	C6—C1—C2—C3	−0.4 (3)
Ni1—N3—C17—C18	177.28 (18)	C6—C1—C7—N2	−1.1 (3)
Ni1—N3—C19—N4	−177.80 (17)	C6—C1—C7—C8	178.7 (2)
Cl1—C3—C4—C5	179.21 (18)	C7—C1—C2—C3	179.8 (2)
O1—C9—C10—C11	179.9 (2)	C7—C1—C6—O2	−0.7 (3)
O1—C9—C10—C15	−0.5 (3)	C7—C1—C6—C5	−178.9 (2)
O3—C13—C14—C15	179.9 (2)	C9—N1—N2—Ni1	0.9 (2)
N1—N2—C7—C1	−176.82 (18)	C9—N1—N2—C7	−173.69 (19)
N1—N2—C7—C8	3.4 (3)	C9—C10—C11—C12	178.4 (2)
N1—C9—C10—C11	−1.4 (3)	C9—C10—C15—C14	−178.5 (2)
N1—C9—C10—C15	178.2 (2)	C10—C11—C12—C13	1.2 (4)
N2—Ni1—O1—C9	0.95 (14)	C11—C10—C15—C14	1.1 (3)
N2—Ni1—O2—C6	10.33 (18)	C11—C12—C13—O3	179.8 (2)
N2—N1—C9—O1	0.0 (3)	C11—C12—C13—C14	−1.0 (4)
N2—N1—C9—C10	−178.63 (17)	C12—C13—C14—C15	0.9 (4)
N3—Ni1—O1—C9	−179.41 (15)	C13—C14—C15—C10	−1.0 (4)
N3—Ni1—O2—C6	−170.01 (17)	C15—C10—C11—C12	−1.2 (3)
N3—C17—C18—N4	0.3 (3)	C16—O3—C13—C12	−175.6 (3)
C1—C2—C3—Cl1	−178.99 (17)	C16—O3—C13—C14	5.3 (4)
C1—C2—C3—C4	−0.8 (4)	C17—N3—C19—N4	−0.1 (3)
C2—C1—C6—O2	179.5 (2)	C18—N4—C19—N3	0.3 (3)
C2—C1—C6—C5	1.3 (3)	C19—N3—C17—C18	−0.2 (3)
C2—C1—C7—N2	178.7 (2)	C19—N4—C18—C17	−0.3 (3)

### Hydrogen-bond geometry (Å, º)

**Table:**

*D*—H···*A*	*D*—H	H···*A*	*D*···*A*	*D*—H···*A*
N4—H4···O2^i^	0.92 (4)	1.93 (4)	2.818 (3)	161 (3)
C8—H8*A*···Cl1^ii^	0.96	2.86	3.700 (3)	147

Symmetry codes: (i) −*y*+5/4, *x*+1/4, −*z*+5/4; (ii) −*x*+3/2, −*y*+1, *z*−1/2.
